# New spatial records of vascular plants in the Azores Archipelago: the PRIBES project and the Azorean Biodiversity Portal (ABP) initiatives - I. São Jorge Island (Azores)

**DOI:** 10.3897/BDJ.14.e167704

**Published:** 2026-01-29

**Authors:** Andrea Petrone, Paulo A.V. Borges, Fernando Pereira, Rui B. Elias

**Affiliations:** 1 University of Azores and Universiy of Bologna ERASMUS student, Angra do Heroísmo, Azores, Portugal University of Azores and Universiy of Bologna ERASMUS student Angra do Heroísmo, Azores Portugal; 2 University of Azores, CE3C—Centre for Ecology, Evolution and Environmental Changes, Azorean Biodiversity Group, CHANGE —Global Change and Sustainability Institute, School of Agricultural and Environmental Sciences, Rua Capitão João d’Ávila, Pico da Urze, 9700-042, Angra do Heroísmo, Azores, Portugal University of Azores, CE3C—Centre for Ecology, Evolution and Environmental Changes, Azorean Biodiversity Group, CHANGE —Global Change and Sustainability Institute, School of Agricultural and Environmental Sciences, Rua Capitão João d’Ávila, Pico da Urze, 9700-042 Angra do Heroísmo, Azores Portugal; 3 IUCN SSC Atlantic Islands Invertebrate Specialist Group, Angra do Heroísmo, Azores, Portugal IUCN SSC Atlantic Islands Invertebrate Specialist Group Angra do Heroísmo, Azores Portugal; 4 IUCN SSC Monitoring Specialist Group, Angra do Heroísmo, Azores, Portugal IUCN SSC Monitoring Specialist Group Angra do Heroísmo, Azores Portugal

**Keywords:** floristic inventory, monitoring, invasive species, vascular plants, Azores, island biogeography, endemism, native species, indeterminate species, introduced species, occurrence data, ecology, São Jorge, habitat diversity

## Abstract

**Background:**

The Azores Archipelago is known for its important natural heritage, yet its ecosystems face a “green tsunami” in the form of numerous exotic and invasive species. This influx has wrought serious biodiversity loss and degradation of ecosystem services, representing one of the greatest threats to conservation across the islands. Originating from accelerated global trade and travel, these invasions impact human activities, public health and economic sectors alike. The PRIBES project intends to contribute to "The Regional Strategy for the Management of Terrestrial and Freshwater Exotic and Invasive Species in the Azores" (PRIBES-LIFE-IP- Estratégia regional para o controlo e prevenção de espécies exóticas invasoras - no âmbito do projeto LIFE IP AZORES NATURA, LIFE17 IPE/PT/000010). Recently, a plan was delivered to the Azorean government that proposes as key strategy: an unified Azores Invasive Species Task Force, a central coordination unit and island‐level focal points defined clear leadership roles for agencies and stakeholders (Axis 1), while stringent pre‐export controls, quarantine measures and risk analyses blocked new arrivals (Axis 2); parallel early‐detection teams and citizen‐science networks screened ports, airports and nurseries and triggered rapid eradication protocols (Axis 3), guided by a tiered framework of eradication, containment, control and mitigation chosen on feasibility and cost–benefit grounds (Axis 4). Simultaneously, national and international partnerships with IUCN (International Union for Conservation of Nature) ISSG (Invasive Species Specialist Group), CABI (Commonwealth Agricultural Bureaux International) and other island regions fostered data exchange (Axis 5), targeted scientific research investigated invasion pathways and management efficacy (Axis 6) and a central observatory consolidated occurrence records and risk assessments (Axis 7). Meanwhile, outreach campaigns, industry training and school programmes rallied public awareness (Axis 8). The AZORES BIOPORTAL (ABP) is a regional e-infrastructure dedicated to the mobilisation, curation and dissemination of biodiversity data from the Azores. It provides centralised data repository for researchers, policy-makers and educators; validated species checklists, including endemic, native and introduced species; integration with national and international biodiversity networks, including PORBIOTA, GBIF and LifeWatch ERIC; and tools for data visualisation and access, supporting conservation, ecological research and environmental management. ABP follows the FAIR (Findable, Accessible, Interoperable, Reusable) and supports open science. Mapping the occurrence of both native (endemic and non endemic) and exotic species is of key importance for the PRIBES project and the ABP intiative.

**New information:**

A total of 243 vascular plant taxa were recorded across São Jorge Island, encompassing 89 families. These records correspond to 4,524 individual plant occurrences, including repeated observations of the same species across different sites. As each photographic observation is tied to unique geographic coordinates, all recorded specimens represent new spatial records for the Island’s flora. Amongst the taxa, 53 are considered endemic to the Azores, 131 are introduced, 58 are native and one species (*Dracaena draco* (L.) L.) is of indeterminate status. These correspond to 1,773 individual occurrences of endemic taxa, 1779 introduced, 970 native and one with indeterminate status. At the family level, 31 families include endemic taxa, 63 include introduced taxa, 34 include native taxa and one family contains a taxon of indeterminate status.

The inventory includes several noteworthy Azorean endemics, spanning both ferns and flowering plants. Amongst the ferns, notable records include Crisped Buckler Fern *Dryopteris crispifolia* Rasbach, Reichst. & Vida, Azorean Buckler Fern *Dryopteris azorica* (Christ) Alston and Azorean Rockcap Fern Polypodium macaronesicum subsp. azoricum (Vasc.) Rumsey, Carine & Robba. Iconic flowering species and woody endemics recorded during the survey comprise Azorean Cherry Prunus lusitanica subsp. azorica (Mouill.) Franco, Azorean Buckthorn *Frangula azorica* Grubov, Azorean Eyebright *Euphrasia grandiflora* Hochst. ex Seub., Azorean Greater-hawkbit *Leontodon filii* (Hochst. ex Seub.) Paiva & Ormonde and Narrow-lipped Butterfly Orchid *Platanthera micrantha* (Hochst. ex Seub.) Schltr. Additional endemic taxa include Azorean Dock *Rumex azoricus* Rech.f., Azorean Holly *Ilex azorica* Gand., Azorean Umbrella Milkwort *Tolpis azorica* (Nutt.) P. Silva and the hemiparasitic Azorean Dwarf Mistletoe *Arceuthobium azoricum* Wiens & Hawksw. Other significant native species recorded include the ferns Wilson's Filmy-fern *Hymenophyllum wilsonii* Hook., Killarney Fern *Vandenboschia speciosa* (Willd.) G.Kunkel and Scaly Tongue-fern *Elaphoglossum hirtum* (Sw.) C.Chr., Cretan Thyme *Thymus caespititius* Brot., Many-stalked Spike-rush *Eleocharis multicaulis* (Sm.) Desv. and the more common native Firetree *Morella faya* (Aiton) Wilbur.

Amongst the most problematic surveyed exotic invasive plant species are the Ginger Lily *Hedychium gardnerianum* Sheppard ex Ker-Gawl., Knotweed *Persicaria capitata* (Buch.-Ham. ex D.Don) H.Gross, Bigleaf Hydrangea *Hydrangea macrophylla* (Thunb.) Ser., Crofton Weed *Ageratina adenophora* (Spreng.) R.M.King & H.Rob., Australian Cheesewood *Pittosporum undulatum* Vent. and the Wandering Jew *Tradescantia fluminensis* Vell., as well as the American Pokeweed *Phytolacca americana* L.

## Introduction

**Figure 1.**
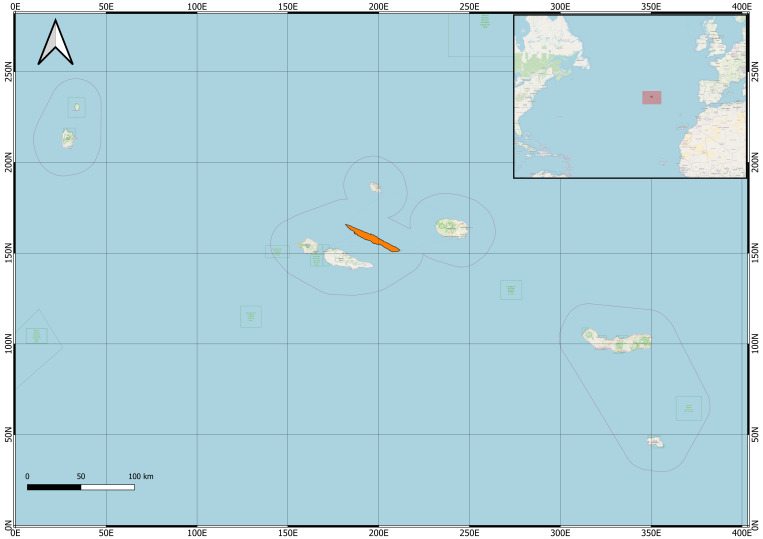
The Azores in the Atlantic Ocean, with São Jorge Island highlighted in orange (credit: Andrea Petrone).

**Figure 2.**
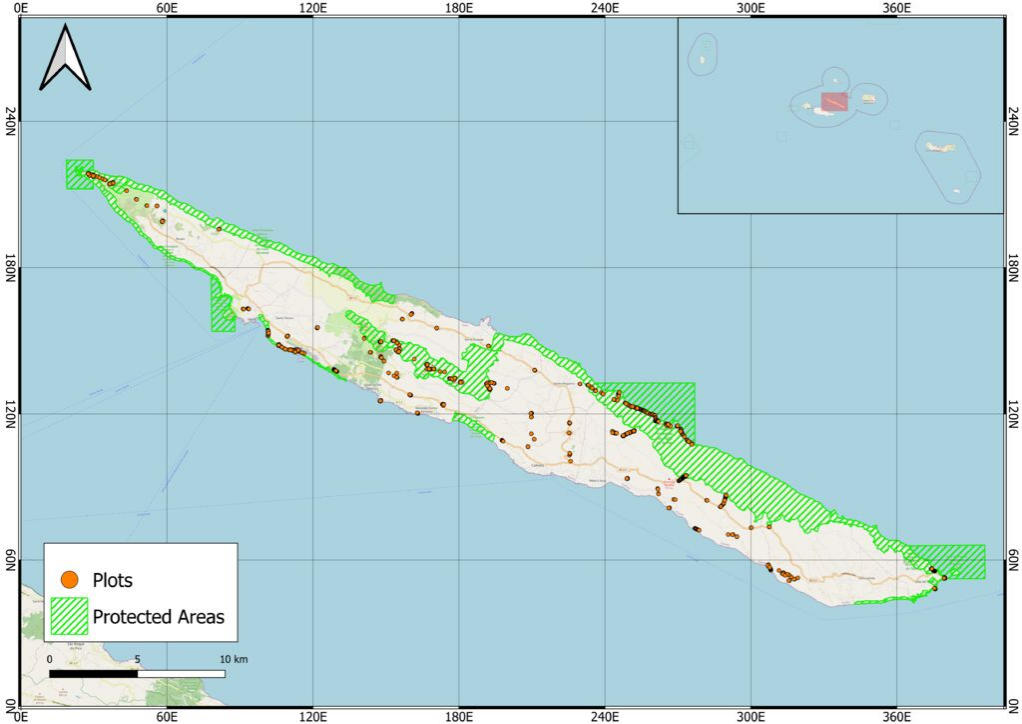
Georeferenced photographic plots (orange points) distributed across São Jorge Island (Azores), primarily within natural and semi-natural habitats. Protected areas are shown in green hatching (data source: UNEP-WCMC and IUCN. (2025), www.protectedplanet.net) (credit: Andrea Petrone).

The Azores Archipelago, located in the North Atlantic Ocean, comprises nine volcanic islands and several islets stretching over 600 km along a northwest-southeast axis (Fig. 1). São Jorge Island (Fig. 2), part of the Central Group, lies at approximately 38°40′09″N, 28°07′19″W and is known for its elongated shape, steep topography and rich ecological variation. It extends approximately 55–56 km in length and 6.5–8 km in width, with an area of about 237–246 km² and reaches its highest elevation at Pico da Esperança (1,053 m a.s.l.) (Madeira and Brum da Silveira 2003). The Island's WNW–ESE orientation results from the alignment of spatter cones along active dextral normal faults, reflecting the tectono-magmatic fabric of the Azores Plateau (Madeira 1998), while its dramatic coastal cliffs — some rising 400–800 m above sea level — and numerous «Fajãs» formed by landslides or lava flows create a highly heterogeneous landscape (Zanon and Viveiros 2019). The climate of the Azores Archipelago is highly maritime-influenced and humid-subtropical, with year-round temperatures generally ranging between 14 and 25°C, although local microclimates can produce marked variation. Winters are mild and rainy, whereas summers are warmer and sunnier, though precipitation may occur at any time of year. High humidity, strong winds and regular rainfall are characteristic features, with climatic conditions strongly shaped by the surrounding Atlantic Ocean and the influence of the Gulf Stream.

São Jorge was once entirely covered by native forests, notably the evergreen laurel forest (Laurisilva), the natural vegetation formation of the warm-temperate zonobiome according to the global vegetation classification by Walter & Breckle (Gloser 2004), a vegetation formation dominant throughout the Azores before human colonisation in the 15^th^ century (Elias et al. 2016). However, anthropogenic pressures, agricultural expansion, infrastructure development and the introduction of exotic species have drastically reduced this native cover, leaving only fragments above 600 m elevation (Elias et al. 2016). Today, less than 10% of the original forest remains, primarily dominated by *Juniperus brevifolia*, *Erica azorica*, *Laurus azorica* and *Ilex azorica*. São Jorge lacks suitable conditions for coastal woodlands due to its steep coastal cliffs, but hosts some of the most extensive areas potentially suitable for *Juniperus–Ilex* montane forests, which could occupy up to 25% of the Island’s surface. These forests typically occur between 600 and 900 m a.s.l. within the upper thermotemperate–hyperhumid bioclimatic zone and are characterised by high rainfall, persistent humidity and the dominance of endemic species such as *Juniperus brevifolia*, *Ilex azorica* and *Laurus azorica* (Elias et al. 2016). Nevertheless, the ecological integrity of the Island is increasingly threatened by invasive species, such as *Pittosporum undulatum*, *Hedychium gardnerianum* and *Carpobrotus edulis*, which can outcompete native flora and alter community structure (Silva and Smith 2004, Borges et al. 2010, Borges Silva et al. 2017, Borges Silva et al. 2018). These invasions are facilitated by human activities, particularly in lowland and accessible «fajã» zones, flat coastal debris fields or lava-delta platforms formed by cliff collapse, coastal erosion or lava flows, a distinctive geomorphological feature of São Jorge (Hildenbrand et al. 2008).

Despite numerous floristic explorations since the 19^th^ century, detailed, spatially-explicit data on plant species occurrences across the varied habitats of São Jorge remain limited. Comprehensive plant inventories such as "A list of the terrestrial and marine biota from the Azores" (Borges et al. 2016) (which includes plant species lists for each island) and "Guia prático da flora nativa dos Açores" (Elias et al. 2024) are crucial for understanding biogeographical patterns, assessing ecological changes and guiding conservation strategies (Whittaker et al. 2005).

This work is part of a broader effort to document and monitor the vascular flora of the Azorean Archipelago within the framework of the PRIBES project and the Azorean Biodiversity Portal (ABP) initiative. The ABP (https://azoresbioportal.uac.pt) (Borges et al. 2010) is a regional e-infrastructure of biodiversity data from the Azores, compiling verified species records, based on historical literature, museum collections and field observations, currently covering approximately 11,500 marine and terrestrial taxa. Species occurrences are georeferenced on a 500 m × 500 m grid and enriched with detailed metadata including colonisation status, conservation information and photographic records. The dataset presented herein provides a new contribution to the ABP, enhancing the spatial resolution of plant distribution data for São Jorge Island and supporting long-term biodiversity monitoring and biogeographical analysis in the region.

## General description

### Purpose

Within the scope of the PRIBES project (see also Borges et al. (2022)), the aim of this study was to develop a spatially-explicit inventory of exotic potentially invasive plant species across diverse natural and semi-natural areas of São Jorge Island (Azores).

### Additional information

This work was also part of a collaboration with the PORBIOTA projet, aiming to map the distribution of Azorean biodiversity in the AZORES BIOPORTAL.

## Project description

### Title

Mapping Vascular Plant Diversity on São Jorge Island under the PRIBES project

### Personnel

The original project was conceived by Rui B. Elias and Paulo A.V. Borges.

Fieldwork (site selection and experimental setting): Rui B. Elias.

Fieldwork (authorisation): Azorean Regional Directorate for the Environment.

Fieldwork: Fernando Pereira and Rui B. Elias.

Taxonomists: Andrea Petrone and Rui B. Elias.

Darwin Core Databases: Andrea Petrone and Paulo A.V. Borges.

### Funding

Direcção Regional do Ambiente - PRIBES (LIFE17 IPE/PT/000010) (2019-2020). PORBIOTA - “ACORES-01-0145-FEDER-000072 - AZORES BIOPORTAL”, funded by the Operational Programme Azores 2020 (85% ERDF and 15% regional funds) (2019-2021). RBE and PAVB are currently funded by FCT through national and European funds by UID/00329/2025 - Centre for Ecology, Evolution and Environmental Changes (CE3C) and this project was also funded by the Regional Directorate for Science, Innovation and Development [Regional Government of the Azores] through the PROSCIENTIA Incentive System (M1.1.A/FUNC.UI&D/021/2025 [UI&D/GBA/2025]).

## Sampling methods

### Sampling description

Fieldwork was conducted over a seven-day period, from 13 July to 19 July 2020. Georeferenced photographs were collected using a Garmin Montana 750i GPS unit, with Multi-GNSS (Global Navigation Satellite System) support and WAAS/EGNOS correction systems that enhance GPS signals with improved accuracy (2-3 m), integrity (error checking) and availability in open-sky conditions. Photographs were taken along an altitudinal and ecological gradient, from coastal areas to high-elevation humid forests, scrublands and grasslands, capturing a variety of habitat types, vegetation density and plant abundance (Fig. 2). Areas with a high degree of anthropogenic disturbance (e.g. intensive pastures, *Cryptomeria* plantations or urban areas) were avoided. The survey took place mainly on natural and semi-natural areas, along trails and road sides, slopes, craters and cliffs. Plant species, visible in the images, were later identified by expert taxonomists with the help of flora guides (e.g. Schaefer (2021)) and online databases (e.g. ABP (2025), Flora-On (2025), POWO (2025)).

Using georeferenced photographs in vegetation surveys provides crucial spatial and ecological information, especially if one needs to survey large areas over a limited amount of time. This approach provides precise geo-locations, enables long-term monitoring and ground truthing (verifying the accuracy of data collected remotely) and is also very important for nature conservation purposes. It can be used for tracking invasive species spread, monitoring post-fire regeneration, document phenology (flowering, fruiting stages) across seasons or recording rare or threatened plant populations with precise location data.

### Quality control

Species taxonomic rank and conservation status were assigned, based on: a) the information available through the Azores Bioportal – PORBIOTA (https://azoresbioportal.uac.pt/pt/) (see also Elias et al. (2024)); b) the official biodiversity platform for the Azores Archipelago, which integrates validated and up-to-date data on the region’s flora (including endemism, invasiveness and habitat preference).

## Geographic coverage

### Description

São Jorge Island in the Azores (Figs 1, 2).

### Coordinates

38.542 and 38.754 Latitude; -28.312 and -27.753 Longitude.

## Taxonomic coverage

### Description

Kingdom: Plantae

Phylum: Tracheophyta.

Class: Cycadopsida, Equisetopsida, Liliopsida, Lycopodiopsida, Magnoliopsida, Pinopsida, Polypodiopsida and Selaginellopsida.

Order: Alismatales, Apiales, Aquifoliales, Asterales, Asparagales, Boraginales, Brassicales, Caryophyllales, Commelinales, Cornales, Cycadales, Cyatheales, Dipsacales, Equisetales, Ericales, Fabales, Fagales, Geraniales, Gentianales, Hymenophyllales, Lamiales, Laurales, Lilliales, Lycopodiales, Malpighiales, Malvales, Myrtales, Osmundales, Pinales, Poales, Polypodiales, Ranunculales, Rosales, Santalales, Saxifragales, Sapindales, Selaginellales, Solanales, Vitales and Zingiberales.

Family: Aizoaceae, Amaranthaceae, Apiaceae, Apocynaceae, Araceae, Araliaceae, Asparagaceae, Asphodelaceae, Aspleniaceae, Athyriaceae, Asteraceae, Basellaceae, Blechnaceae, Boraginaceae, Brassicaceae, Campanulaceae, Cactaceae, Cannaceae, Caryophyllaceae, Commelinaceae, Convolvulaceae, Crassulaceae, Culcitaceae, Cyatheaceae, Cycadaceae, Cyperaceae, Dennstaedtiaceae, Dipsacaceae, Dryopteridaceae, Equisetaceae, Ericaceae, Euphorbiaceae, Fabaceae, Frankeniaceae, Gentianaceae, Geraniaceae, Hydrangeaceae, Hymenophyllaceae, Hypericaceae, Iridaceae, Juncaceae, Lauraceae, Lamiaceae, Liliaceae, Lycopodiaceae, Lythraceae, Malvaceae, Moraceae, Musaceae, Myricaceae, Myrsinaceae, Myrtaceae, Nephrolepidaceae, Nyctaginaceae, Onagraceae, Orobanchaceae, Orchidaceae, Osmundaceae, Papaveraceae, Phytolaccaceae, Pinaceae, Pittosporaceae, Plantaginaceae, Poaceae, Polygonaceae, Polypodiaceae, Primulaceae, Pteridaceae, Rhamnaceae, Ranunculaceae, Rosaceae, Rubiaceae, Ruppiaceae, Salicaceae, Santalaceae, Scrophulariaceae, Selaginellaceae, Simaroubaceae, Solanaceae, Thelypteridaceae, Tropaeolaceae, Verbenaceae, Violaceae, Vitaceae and Zingiberaceae.

## Temporal coverage

**Data range:** 2020-7-13 – 2020-7-19.

## Usage licence

### Usage licence

Creative Commons Public Domain Waiver (CC-Zero)

## Data resources

### Data package title

Mapping Vascular Plant Diversity on São Jorge Island under the PRIBES project

### Resource link

https://doi.org/10.15468/drbja9

### Alternative identifiers

https://www.gbif.org/dataset/9e53efa1-18a2-429f-8411-17d273bfc479

### Number of data sets

2

### Data set 1.

#### Data set name

Event Table

#### Data format

Darwin Core Archive format

#### Character set

UTF-8

#### Download URL

http://ipt.gbif.pt/ipt/resource?r=sao_jorge_plants

#### Data format version

1.1

#### Description

The dataset was published in the Global Biodiversity Information Facility platform, GBIF (Petrone et al. 2025). The following data-table includes all the records for which a taxonomic identification of the species was possible. The dataset submitted to GBIF is structured as a sample event dataset that has been published as a Darwin Core Archive (DwCA), which is a standardised format for sharing biodiversity data as a set of one or more data tables. The core data file contains 1655 records (eventID). This GBIF IPT (Integrated Publishing Toolkit, Version 2.5.6) archives the data and, thus, serves as the data repository. The data and resource metadata are available for download in the Portuguese GBIF Portal IPT (Petrone et al. 2025).

**Data set 1.** 

Column label	Column description
eventID	Identifier of the event, unique for the dataset.
datasetName	The name identifying the dataset from which the record was derived.
continent	The name of the continent in which the dcterms:Location occurs.
stateProvince	The name of the next smaller administrative region than country (state, province, canton, department, region etc.) in which the dcterms:Location occurs.
islandGroup	The name of the island group in which the dcterms:Location occurs.
island	The name of the island on or near which the dcterms:Location occurs.
country	The name of the country or major administrative unit in which the dcterms:Location occurs.
countryCode	The standard code for the country in which the dcterms:Location occurs.
municipality	The full, unabbreviated name of the next smaller administrative region than county (city, municipality etc.) in which the dcterms:Location occurs. Do not use this term for a nearby named place that does not contain the actual dcterms:Location.
minimumElevationInMetres	The lower limit of the range of elevation (altitude, usually above sea level), in metres.
decimalLongitude	The geographic longitude (in decimal degrees, using the spatial reference system given in dwc:geodeticDatum) of the geographic centre of a dcterms:Location. Positive values are east of the Greenwich Meridian, negative values are west of it. Legal values lie between -180 and 180, inclusive.
decimalLatitude	The geographic latitude (in decimal degrees, using the spatial reference system given in dwc:geodeticDatum) of the geographic centre of a dcterms:Location. Positive values are north of the Equator, negative values are south of it. Legal values lie between -90 and 90, inclusive.
habitat	Description of the habitat where the specimen was found.
geodeticDatum	The spatial reference system upon which the geographic coordinates are based.
coordinateUncertaintyInMetres	The horizontal distance (in metres) from the given dwc:decimalLatitude and dwc:decimalLongitude describing the smallest circle containing the whole of the dcterms:Location. Leave the value empty if the uncertainty is unknown, cannot be estimated or is not applicable (because there are no coordinates). Zero is not a valid value for this term.
coordinatePrecision	A decimal representation of the precision of the coordinates given in the dwc:decimalLatitude and dwc:decimalLongitude.
georeferenceSources	A list (concatenated and separated) of maps, gazetteers or other resources used to georeference the dcterms:Location, described specifically enough to allow anyone in the future to use the same resources.
day	The integer day of the month on which the dwc:Event occurred.
month	The integer month in which the dwc:Event occurred.
year	The four-digit year in which the dwc:Event occurred, according to the Common Era Calendar.
eventDate	The date-time or interval during which a dwc:Event occurred. For occurrences, this is the date-time when the dwc:Event was recorded. Not suitable for a time in a geological context.
sampleSizeValue	A numeric value for a measurement of the size (time duration, length, area or volume) of a sample in a sampling dwc:Event.
sampleSizeUnit	The unit of measurement of the size (time duration, length, area or volume) of a sample in a sampling dwc:Event.
eventRemarks	Comments or notes about the dwc:Event.
samplingProtocol	The names of, references to or descriptions of the methods or protocols used during a dwc:Event.

### Data set 2.

#### Data set name

Occurrence Table

#### Data format

Darwin Core Archive format

#### Character set

UTF-8

#### Download URL

http://ipt.gbif.pt/ipt/resource?r=sao_jorge_plants

#### Data format version

1.1

#### Description

The dataset was published in the Global Biodiversity Information Facility platform, GBIF (Petrone et al. 2025). The following data table includes all the records for which a taxonomic identification of the species was possible. The dataset submitted to GBIF is structured as an occurrence table that has been published as a Darwin Core Archive (DwCA), which is a standardised format for sharing biodiversity data as a set of one or more data tables. The core data file contains 4523 records (occurrenceID). This GBIF IPT (Integrated Publishing Toolkit, Version 2.5.6) archives the data and, thus, serves as the data repository. The data and resource metadata are available for download in the Portuguese GBIF Portal IPT (Petrone et al. 2025).

**Data set 2.** 

Column label	Column description
eventID	An identifier for the set of information associated with a dwc:Event (something that occurs at a place and time). May be a global unique identifier or an identifier specific to the dataset.
type	The nature of the resource.
licence	A legal document giving official permission to do something with the resource.
institutionID	The identifier for the institution having custody of the object or information referred to in the record.
institutionCode	The acronym of the institution having custody of the object or information referred to in the record.
occurrenceID	An identifier for the dwc:Occurrence (as opposed to a particular digital record of the dwc:Occurrence). In the absence of a persistent global unique identifier, construct one from a combination of identifiers in the record that will most closely make the dwc:occurrenceID globally unique.
basisOfRecord	The specific nature of the data record.
dynamicProperties	A list of additional measurements, facts, characteristics or assertions about the record. Meant to provide a mechanism for structured content.
establishmentMeans	Statement about whether a dwc:Organism has been introduced to a given place and time through the direct or indirect activity of modern humans.
recordedBy	A list (concatenated and separated) of names of people, groups or organisations responsible for recording the original dwc:Occurrence. The primary collector or observer, especially one who applies a personal identifier (dwc:recordNumber), should be listed first.
identifiedBy	A person, group or organisation who assigned the dwc:Taxon to the subject.
dateIdentified	The date on which the subject was determined as representing the dwc:Taxon.
scientificName	The full scientific name, with authorship and date information, if known. When forming part of a dwc:Identification, this should be the name in lowest level taxonomic rank that can be determined. This term should not contain identification qualifications, which should instead be supplied in the dwc:identificationQualifier term.
kingdom	The full scientific name of the kingdom in which the dwc:Taxon is classified.
phylum	The full scientific name of the phylum or division in which the dwc:Taxon is classified.
class	The full scientific name of the class in which the dwc:Taxon is classified.
order	The full scientific name of the order in which the dwc:Taxon is classified.
family	The full scientific name of the family in which the dwc:Taxon is classified.
genus	The full scientific name of the genus in which the dwc:Taxon is classified.
specificEpithet	The name of the first or species epithet of the dwc:scientificName.
infraspecificEpithet	The name of the lowest or terminal infraspecific epithet of the dwc:scientificName, excluding any rank designation.
taxonRank	The taxonomic rank of the most specific name in the dwc:scientificName.
scientificNameAuthorship	The authorship information for the dwc:scientificName formatted according to the conventions of the applicable dwc:nomenclaturalCode.

## Additional information

**Figure 3.**
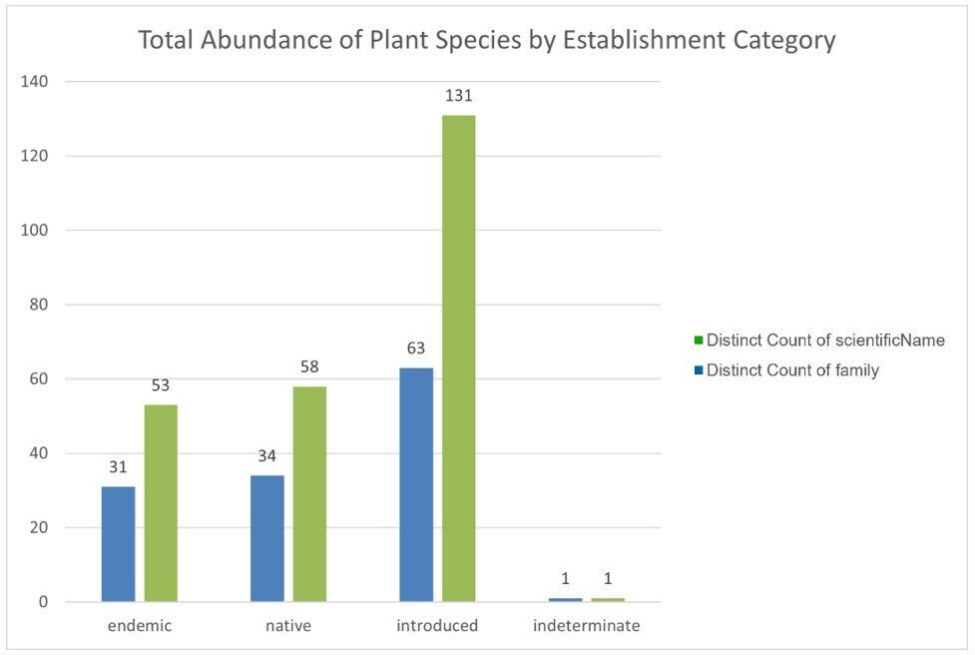
Number of distinct vascular plant species (scientificName) and families (family) recorded on São Jorge Island, categorised by biogeographic status (endemic, introduced, native, indeterminate).

**Figure 4.**
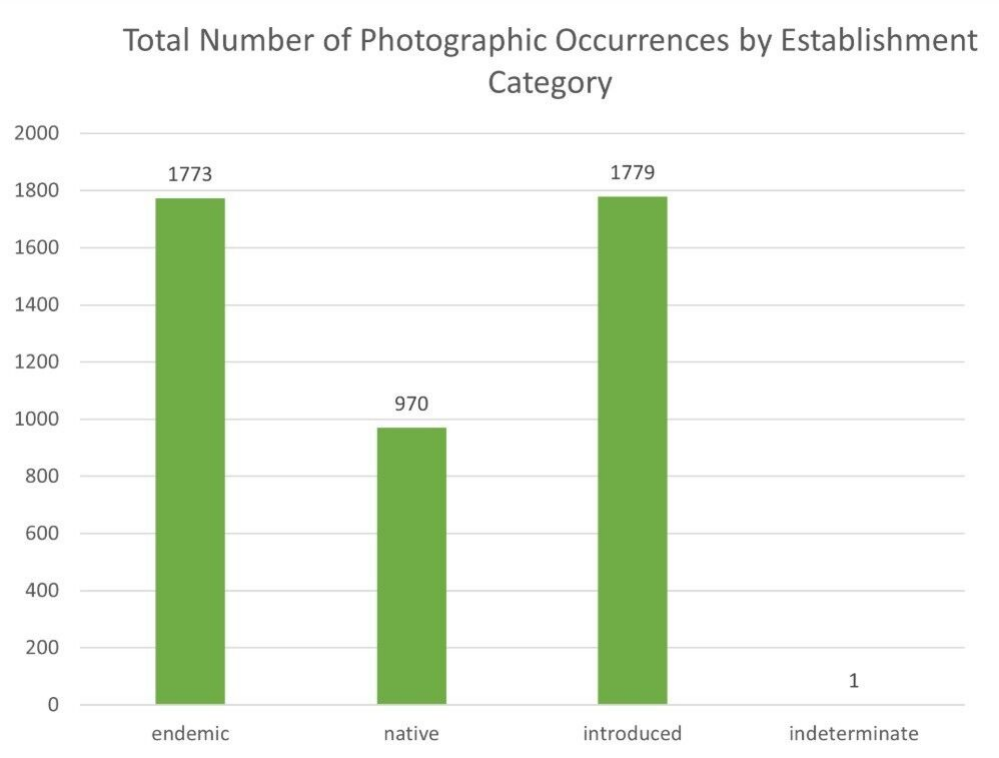
Total number of photographic occurrences of vascular plant species per biogeographic status, reflecting relative abundance across surveyed locations.

A comprehensive survey of São Jorge Island’s vascular flora yielded 243 distinct taxa, representing eight classes, 40 orders and 89 families (Suppl. material 1). In total, 4524 photographic records — each georeferenced to unique coordinates — were obtained, thereby providing entirely new spatial data for every documented species. Of these 243 taxa, 53 (1774 occurrences) are Azorean endemics, 58 (970 occurrences) are native non-endemics, 131 (1779 occurrences) are introduced and one (*Dracaena draco* (L.) L.; 1 occurrence) remains of uncertain status. At the family level, endemics occur in 31 families, natives in 34, introduced taxa in 63 and one family contains the single taxon of indeterminate provenance (Figs 3, 4).

**Figure 5.**
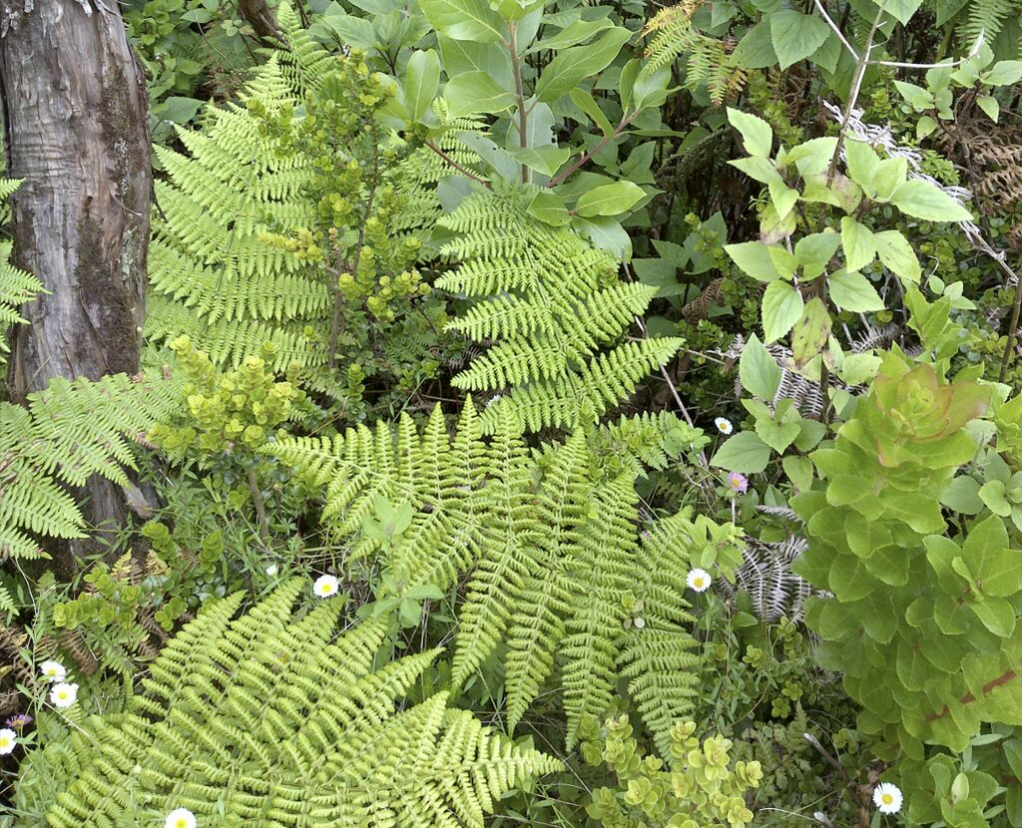
Fronds of the endemic fern *Dryopteris azorica* (Christ) Alston (credit: Rui B. Elias).

**Figure 6.**
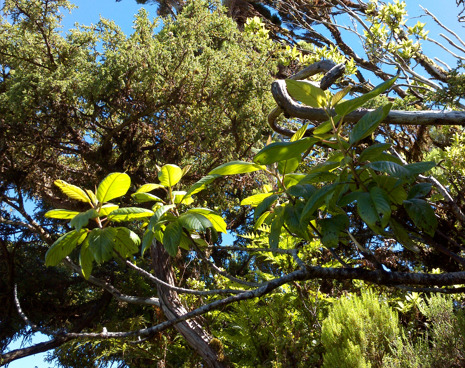
The endemic *Frangula azorica* Grubov (credit: Rui B. Elias).

**Figure 7.**
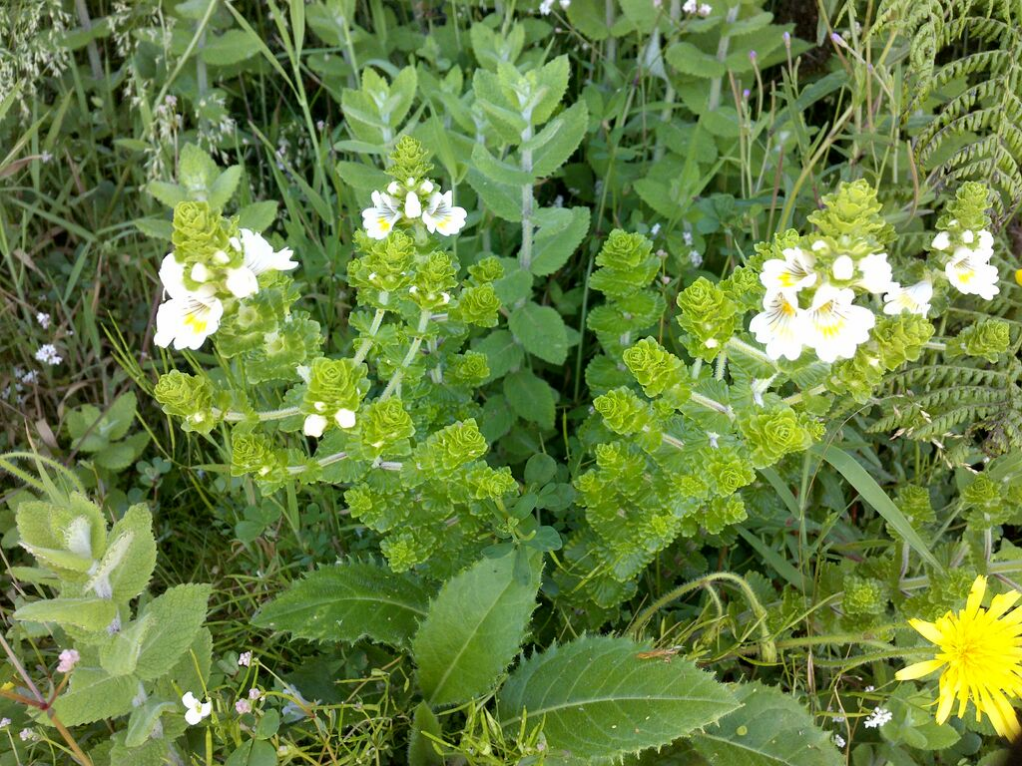
The endemics *Euphrasia grandiflora* Hochst. ex Seub. and *Leontodon filii* (Hochst. ex Seub.) Paiva & Ormonde, along with the introduced *Mentha suaveolens* Ehrh. (credit: Rui B. Elias).

**Figure 8.**
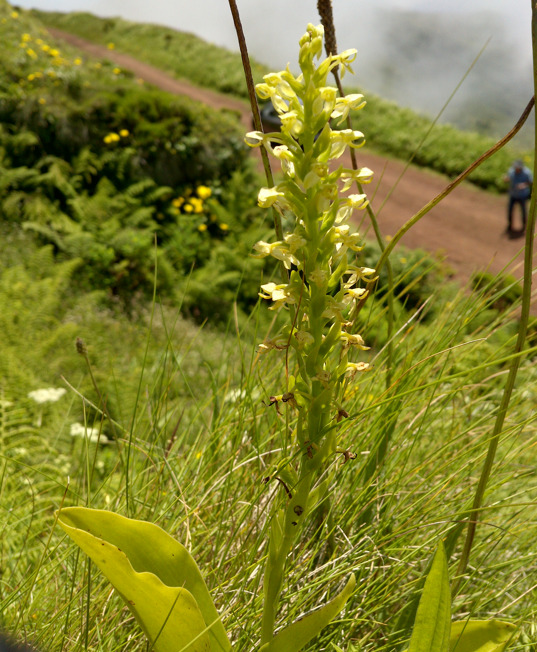
The endemic *Platanthera micrantha* (Hochst. ex Seub.) Schltr. (credit: Rui B. Elias).

**Figure 9.**
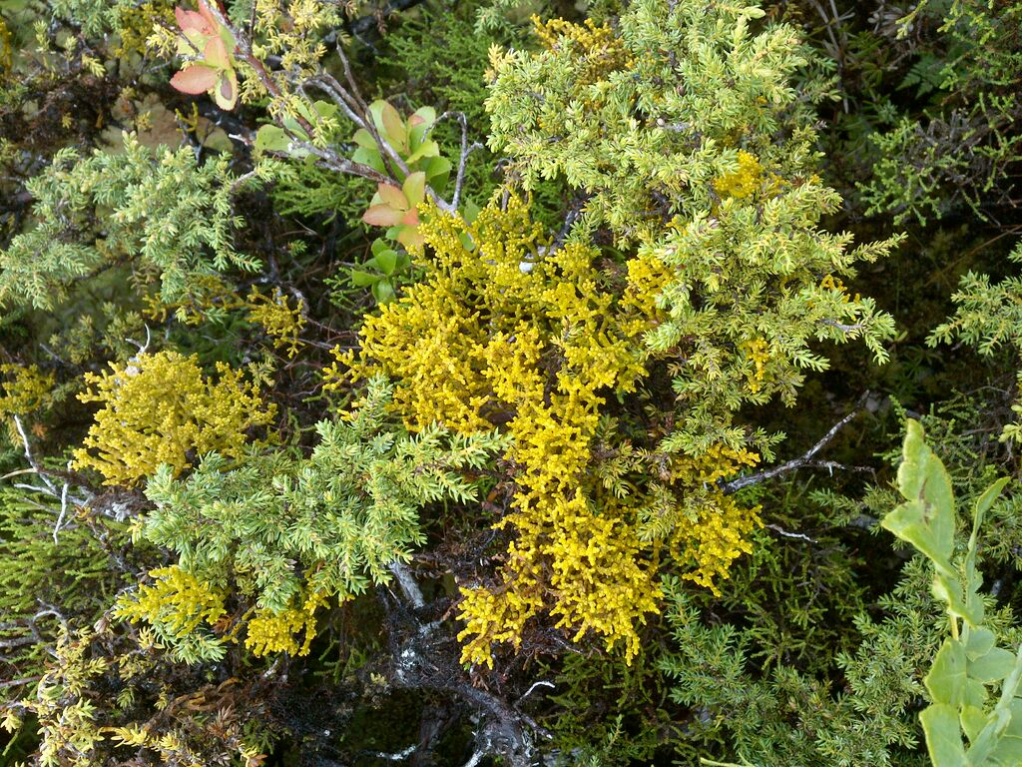
The endemic hemiparasitic *Arceuthobium azoricum* Wiens & Hawksw. parasitising Juniperus brevifolia (Hochst. ex Seub.) Antoine subsp. brevifolia and growing close to the species *Vaccinium cylindraceum* Sm. (credit: Rui B. Elias).

The inventory includes several notable endemics, such as the ferns *Dryopteris crispifolia* Rasbach, Reichst. & Vida, *Dryopteris azorica* (Christ) Alston (Fig. 5) and Polypodium macaronesicum subsp. azoricum (Vasc.) Rumsey, Carine & Robba, along with iconic flowering species and shrubs, such as Prunus lusitanica subsp. azorica (Mouill.) Franco, *Frangula azorica* Grubov (Fig. 6), *Euphrasia grandiflora* Hochst. ex Seub., *Leontodon filii* (Hochst. ex Seub.) Paiva & Ormonde (Fig. 7), *Platanthera micrantha* (Hochst. ex Seub.) Schltr. (Fig. 8), *Rumex azoricus* Rech.f., *Ilex azorica* Gand., *Tolpis azorica* (Nutt.) P. Silva and the hemiparasitic *Arceuthobium azoricum* Wiens & Hawksw (Fig. 9)

Non-native species make up a substantial portion of our records, many of which are recognised invaders that significantly alter native plant communities. Amongst the most problematic are the ginger lily *Hedychium gardnerianum* Sheppard ex Ker-Gawl., knotweed *Persicaria capitata* (Buch.-Ham. ex D.Don) H.Gross, bigleaf hydrangea *Hydrangea macrophylla* (Thunb.) Ser., crofton weed *Ageratina adenophora* (Spreng.) R.M.King & H.Rob., Australian cheesewood *Pittosporum undulatum* Vent. and the wandering Jew *Tradescantia fluminensis* Vell., as well as the American pokeweed *Phytolacca americana* L. — all frequently observed forming dense stands that suppress indigenous understory species and disrupt successional trajectories.

## Supplementary Material

2646FE87-1857-5889-A430-C43A7DD1529B 10.3897/BDJ.14.e167704.suppl1 Supplementary material 1 Table with the list of species Data type taxonomic list Brief description This supplementary table provides the complete list of vascular plant taxa recorded in the study, including their colonisation status and, for each species or subspecies, the corresponding number of occurrences, presented in the column 'Count', giving an idea of how widespread the taxon is on the Island. File: oo_1387343.csv https://binary.pensoft.net/file/1387343 Andrea Petrone, Paulo A.V. Borges, Fernando Pereira, Rui B. Elias
